# Supplementary material for: Dynamic transcriptomic profiles of zebrafish gills in response to zinc supplementation
Source: BMC Genomics. 2010 Oct 11;11:553. doi: 10.1186/1471-2164-11-553 (PMC3091702; doi:10.1186/1471-2164-11-553)
Supplement: Additional file 2 — Interactive Direct Interaction Network representing the molecular interactions between zinc, copper, iron, calcium and proteins encoded by transcripts changed by zinc supplementation. Mini web-site containing index.html and hyperlinked pages in subdirectory describing a Direct Interaction Network automatically generated based on curated interactions contained within the proprietary PathwayArchitect database. Ovals represent proteins and the circles symbolize metal ions. Objects are coloured by their abundance in zebrafish at the time-point they were significantly different from the control is a scale from -4 fold (dark green) to +4 fold (dark red). Where significant differences were found at more than one time-point, the colour overlay shows expression at the first instance. Dark blue squares denote 'binding', and light blue squares 'expression'; green squares stand for 'regulation', green diamonds for 'metabolism', and green circles for 'promoter binding'. Arrow heads indicate directionality of the interaction where annotated. All nodes and edges can be further interrogated by selecting the relative area of the image. [file 1471-2164-11-553-S2.zip › PathwayArchitect Zn xs DIN/754134.html]

# REGULATION:

|  |  |
| --- | --- |
| Type | REGULATION |
| Effect | Positive |


---

|  |  |
| --- | --- |
| Score | 0 |


---

|  |  |
| --- | --- |
| Reference Count | 3 |


---

|  |  |
| --- | --- |
| Mechanism | Unknown |


---

|  |  |
| --- | --- |
| Reference:0 || Sentence | "The effects of iron chelators, metabolic inhibitors and the cellular activators, lipopolysaccharide (LPS) and tumour necrosis factor-alpha (TNF-alpha), on Tf and Fe uptake were investigated." |
| PMID | 10865941 |
| Year | 2000 |
| Species | Mouse |
| Journal | Endothelium |
| RefScore | 0 |
| Source | PArchNLP |
  |
|  |  |


---

|  |  |
| --- | --- |
 Reference:1 || Sentence | "The iron load caused a 6.5-fold increase in the mucosal Fe-concentration in the proximal as well as distal colon." |
| PMID | 3409203 |
| Year | 1988 |
| Species | Mouse |
| Journal | Cancer Lett |
| RefScore | 1 |
| Source | PArchNLP |
  ||  |  |


---

|  |  |
| --- | --- |
 Reference:2 || Sentence | "Third, iron release and IRP-1 activation were observed in lysates from control or iron-loaded macrophages, containing increasing levels of Fe-S clusters, but not in lysates from iron-starved macrophages, in which aconitase had already undergone cluster disassembly and switched to IRP-1. \*NO was less efficient than ONOO(-) in attacking the Fe-S cluster of cytoplasmic aconitase; in fact, SIN-1-dependent iron release and IRP-1 activation were diminished by superoxide dismutase, which scavenged O(2)(\*-) before it reacted with \*NO to form ONOO(-)." |
| PMID | 12044177 |
| Year | 2002 |
| Species | Mouse |
| Journal | Biochemistry |
| RefScore | 2 |
| Source | PArchNLP |
  |


---

|  |  |
| --- | --- |
